# Applicability of an Automated Model and Parameter Selection in the Prediction of Screening-Level PTSD in Danish Soldiers Following Deployment: Development Study of Transferable Predictive Models Using Automated Machine Learning

**DOI:** 10.2196/17119

**Published:** 2020-07-22

**Authors:** Karen-Inge Karstoft, Ioannis Tsamardinos, Kasper Eskelund, Søren Bo Andersen, Lars Ravnborg Nissen

**Affiliations:** 1 Research and Knowledge Centre The Danish Veterans Centre Ringsted Denmark; 2 Department of Psychology University of Copenhagen Copenhagen Denmark; 3 Department of Computer Science University of Crete Heraklion, Crete Greece; 4 Gnosis Data Analysis PC Heraklion Greece; 5 Department of Military Psychology The Danish Veterans Centre Copenhagen Denmark

**Keywords:** decision support, machine learning, mental health, PTSD, military, screening

## Abstract

**Background:**

Posttraumatic stress disorder (PTSD) is a relatively common consequence of deployment to war zones. Early postdeployment screening with the aim of identifying those at risk for PTSD in the years following deployment will help deliver interventions to those in need but have so far proved unsuccessful.

**Objective:**

This study aimed to test the applicability of automated model selection and the ability of automated machine learning prediction models to transfer across cohorts and predict screening-level PTSD 2.5 years and 6.5 years after deployment.

**Methods:**

Automated machine learning was applied to data routinely collected 6-8 months after return from deployment from 3 different cohorts of Danish soldiers deployed to Afghanistan in 2009 (cohort 1, N=287 or N=261 depending on the timing of the outcome assessment), 2010 (cohort 2, N=352), and 2013 (cohort 3, N=232).

**Results:**

Models transferred well between cohorts. For screening-level PTSD 2.5 and 6.5 years after deployment, random forest models provided the highest accuracy as measured by area under the receiver operating characteristic curve (AUC): 2.5 years, AUC=0.77, 95% CI 0.71-0.83; 6.5 years, AUC=0.78, 95% CI 0.73-0.83. Linear models performed equally well. Military rank, hyperarousal symptoms, and total level of PTSD symptoms were highly predictive.

**Conclusions:**

Automated machine learning provided validated models that can be readily implemented in future deployment cohorts in the Danish Defense with the aim of targeting postdeployment support interventions to those at highest risk for developing PTSD, provided the cohorts are deployed on similar missions.

## Introduction

Posttraumatic stress disorder (PTSD) is a relatively common problem following exposure to trauma [1]. Following deployment to war zones, PTSD or symptoms thereof are seen in a significant percentage of soldiers. A recent review found average PTSD rates of 12.9% (95% CI 11.3%-14.4%) for military personnel deployed to Iraq and 7.1% (95% CI 4.6%-9.6%) for personnel deployed to Afghanistan among military personnel from the United States, the United Kingdom, and Canada [2]. Among Danish soldiers, cohort studies have found that approximately 10% experience severe symptoms of PTSD 2.5 years after returning from deployment to Afghanistan [3]. With a total of 9949 Danish soldiers deployed to Afghanistan as of December 31, 2018 and 33,131 deployed to different combat zones including Afghanistan, Iraq, and the Balkans, this poses a significant public health problem.

Preventing large-scale bouts of PTSD and the derivative effects among previously deployed soldiers calls for reliable screening tools that can be applied early relative to deployment [4]. However, previous efforts at mental health screening among soldiers before deployment and shortly after returning have so far proved futile [5]. As such, research efforts to date have not enabled primary prevention, where highly vulnerable individuals with a high risk of developing PTSD following deployment are not deployed, or secondary prevention, where early treatment is offered to those in need, best preventing them from developing severe or chronic PTSD [6].

One reason for this lack of success might be the use of traditional statistics when investigating and integrating risk factors into predictive models of postdeployment PTSD [7]. Such models may not be able to account for the multidimensionality and nonlinearity of interacting risk factors for PTSD [8]; as such, they fail to provide an accurate prediction. In recent years, methods of machine learning (ML) have made their way into the literature on trauma reactions and in psychiatry more generally, with the primary aim of early prediction of psychological problems or psychiatric diagnoses such as PTSD [9,10]. ML is a broad term covering computational methods that work by learning from data with the aim of building models that are able to recognize patterns, distinguish between categories, or predict the level or degree of some trait or characteristic [11]. In the specific case of supervised ML, an algorithm is trained in relation to some outcome of interest, with the aim of categorizing individuals as belonging to one or another predefined category [12].

A recent review described 15 studies applying ML methods to predict PTSD or categorize individuals as PTSD cases or noncases [8] and found that, in general, ML prediction models applied to the domain of PTSD prediction reveal promising results. Most of the papers included in the review were cross-sectional (both predictor values and the outcome were measured at the same time point); hence, they were not predictive, but diagnostic, of PTSD status. In general, these cross-sectional studies classified PTSD with very high accuracy (area under the receiver operating characteristic [ROC] curve [AUC] ranging from 0.79 to 0.97). Another group of papers in the review predicted PTSD at a follow-up of <1 year; in general, the prediction accuracies of these studies are respectable. For example, Saxe et al [13] and Rosellini et al [14] both aimed to predict PTSD at 3 months after trauma or release from the hospital, respectively, and did so with high accuracy (AUC=0.79). Finally, three papers in the review predicted PTSD at a follow-up of >1 year, 2 with a follow-up at 15 months [15,16] and one with follow-up at 2.5 years [17]. All three studies achieved acceptable to high accuracy (AUCs ranging from 0.75 to 0.88). While these results are promising, it is clear that more ML prediction studies with longer follow-ups are needed to test the applicability of such methods in practice. Further, focus on future efforts within ML prediction of PTSD should be on how the trained and tested algorithms can be implemented in clinical contexts and for screening purposes.

In the military context, Kessler and colleagues [18] applied ML techniques to predict suicide after hospitalization with psychiatric diagnoses in service members, whereas Rosellini and colleagues [19] used ML to predict postdeployment psychiatric disorder symptoms and interpersonal violence during deployment based on predeployment characteristics. Both studies found that ML provides relatively accurate prediction of the targeted outcomes, with the best performing predictive models in the study by Rosellini et al [19] significantly outperforming logistic regression models. In an earlier study by our own group, we applied a specific ML algorithm, namely support vector machine (SVM), in a cohort study with Danish soldiers deployed to Afghanistan with the aim of predicting PTSD symptomatology 2.5 years after deployment based on predeployment and early postdeployment characteristics [17]. Briefly, we found that long-term posttraumatic stress could be predicted with good accuracy by predeployment indicators (AUC=0.84) and by predeployment indicators combined with indicators collected immediately postdeployment (AUC=0.88).

These studies applied ML to predict PTSD using a variety of ML methods, each requiring expertise and resources for the selection of appropriate algorithms and tuning of hyperparameters. Automated machine learning (AutoML) is a quickly rising subfield of ML promising to ease the application while ensuring correct and optimal utilization of ML methods [20]. Here, we use and test AutoML as implemented in the Just Add Data Bio (JADBio) tool [21]. In brief, JADBio optimizes the final model over a wealth of combinations of feature selection and classification algorithm combinations, along with their hyperparameter values, and estimates the predictive performance of the best-found model.

For prediction models to be applicable across clinical contexts, they must perform well when applied to data and populations of slightly different distributions than the one they were trained on [22]. However, this has not been tested in previous studies using ML to predict PTSD. Further, as already mentioned, few studies have aimed to predict PTSD symptoms over the long term (ie, several years) after traumatic events or military deployment. In this study, we aimed to address this by utilizing data routinely collected from 3 different cohorts deployed to Afghanistan with the Danish Defense that were followed for 2.5 and 6.5 years after returning from deployment. Specifically, in 3 experiments, we test if AutoML, as implemented in JADBio, provides reliable performance estimates; if predictive models trained on one deployment cohort can be used to predict future screening-level PTSD in another deployment cohort; and how accurately we can predict screening-level PTSD 2.5 and 6.5 years after returning home using routinely collected questionnaire data. For the third aim, we report the model type that predicts the outcome best as well as the features selected as most predictive.

## Methods

### Population

The study population in this project included 3 different deployment cohorts that were similar in many regards in that all 3 cohorts were deployed to the same area in Afghanistan, each for a period of approximately 6 months between 2009 and 2013. However, the cohorts were different in some regards too, in that the mission purpose and level of threat were different across the deployments. All 3 deployment cohorts were part of the International Security Assistance Force (ISAF). In this study, cohort 1 (N=287 or N=261 depending on the timing of the outcome assessment, explained later) refers to ISAF7, who were deployed from February 2009 to August 2009; cohort 2 (N=352) refers to ISAF10, who were deployed from August 2010 to February 2011; and cohort 3 (N=232) refers to ISAF15, who were deployed from February 2013 to August 2013. Of note, cohort 1 is a subcohort of that used by Karstoft et al [17]; however, the data are different. Here, we used a new set of predictor data that was collected routinely for all 3 cohorts, but which was not part of the 2015 analysis. Descriptive statistics of the 3 cohorts can be seen in Table 1. In all 3 cohorts, most participants were male (>90%), and the mean age was 30.6-31.3 years. Prior deployment had occurred for 55.0%-63.6% of the cohorts, and 47.6%-59.2% of the cohorts had a military rank of private. Finally, the proportion with screening-level PTSD symptoms was 7.8%-10.0% 6 months after returning home, and 21.7%-27.3% were assessed as having PTSD 2.5 and 6.5 years after deployment.

**Table 1:** Descriptive statistics of the 3 cohorts.

Characteristics	Cohort 1^a^ (N=261), n (%)	Cohort 2 (N=352), n (%)	Cohort 3 (N=232), n (%)
Age (years)^b^	30.6 (8.2)	31.3 (9.9)	31.1 (8.7)
Gender (female)	20 (7.7)	21 (6.0)	19 (8.2)
Previously deployed (yes)	162 (62.3)	193 (55.0)	147 (63.6)
Military rank (private)	154 (59.2)	193 (55.0)	110 (47.6)
Screening-level PTSD^c^ at 6 months	22 (8.5)	35 (10.0)	18 (7.8)
Screening-level PTSD at the 2.5-year or 6.5-year follow-up	71 (27.3)	76 (21.7)	54 (23.4)

^a^Descriptive statistics for cohort 1 are based on the sample who provided outcome data at 6.5 years. Minor differences might be observed in the sample providing outcome data at 2.5 years.

^b^mean (SD).

^c^PTSD: posttraumatic stress disorder.

### Data Material

#### Outcome

The predicted outcome was screening-level PTSD*.* For all 3 cohorts, this was assessed using the civilian version of the PTSD checklist (PCL-C) [23]. The PCL-C contains 17 items mirroring the symptoms of PTSD as defined in the Diagnostic and Statistical Manual of Mental Disorders, fourth edition [24]. Our group has previously validated cutoff scores for the PCL-C in a military population and found that a score ≥44 identifies individuals with severe PTSD symptoms that indicates a likely PTSD diagnosis, while a score ≥30 can be used to identify individuals with moderate or screening-level PTSD [25]. For this study, we applied the cutoff score of 30 since the aim was not to identify individuals that most likely have a diagnosis but to screen for individuals that might be in need of help due to some elevation of symptomatology. For cohort 1, the outcome was assessed 2.5 years and 6.5 years after returning home. For cohort 2, the outcome was assessed 6.5 years after returning home only, while for cohort 3, it was assessed 2.5 years after returning home only. For the aim of this study, we combined the 3 cohorts in the following ways: cohorts 1 and 3 were combined (cohort 1&3_2.5, N=519) with the aim of predicting PTSD 2.5 years after deployment, while cohorts 1 and 2 were combined (cohort 1&2_6.5, N=613) with the aim of predicting PTSD 6.5 years after deployment.

#### Predictors

Predictors for the current project were retrieved from a database containing responses to the Psychological Reactions to International Missions (PRIM) questionnaire, which has been routinely distributed since 1998 to all Danish soldiers 6 months after return from an international deployment with the Danish Defense. The questionnaire contains 125 individual items covering deployment experiences (reported at 6 months after returning home), postdeployment reactions, and postdeployment support as well as 5 validated scales: PTSD symptoms [26], depression symptoms [27], perceived danger [28], witnessing of war atrocities during deployment [28], and postdeployment social support [29]. A list of all items in the PRIM questionnaire, translated into English (Multimedia Appendix 1), as well as their descriptive statistics including level of missingness (Multimedia Appendix 2 and Multimedia Appendix 3) can be seen in the supplementary material. Of note, the level of missingness was <2% for all items except two, which had levels <3%.

### Predictive Modeling With JADBio

For the predictive modeling in this project, we employed the AutoML program JADBio. JADBio has been employed in several other fields to produce novel scientific results (eg, nanomaterial property predictions [21], suicide prediction [30], speech classification [31], bank failure prediction [32], function protein prediction [33], and breast cancer prognosis and drug response prediction [34]). JADBio includes algorithms that are also appropriate for small-sample, high-dimensional biological data, hence the Bio part of the name, but can analyze any type of data that is in a 2-dimensional matrix format, as indicated by the examples provided. Internally, the system employs an artificial intelligence (AI) subsystem that encodes statistical knowledge to select the most appropriate algorithms for transformations, imputation of missing values, feature selection, and predictive modeling, as well as reasonable values for hyperparameters of these algorithms [21]. These selections are fed into what is called the Configuration Generator: Each configuration is a pipeline comprised of algorithms for transformations of features, imputation of missing values, feature selection, and modeling and corresponding hyperparameter values for each algorithm. Thus, a configuration accepts the data matrix and performs all steps necessary to generate a predictive model instance. Based on the choices of the AI system, the Configurator Generator searches in the space of possible configurations to identify one that is optimal, namely, the one that produces, on average, the best performing model instances. An important part of each configuration is the feature selection step, which is based on the statistical equivalent signatures algorithm [35]. The statistical equivalent signatures algorithm aims at identifying multiple feature subsets with the properties that they are of minimal size and optimally predictive for the target. Notice that multiple feature subsets may be equally predictive because of correlations among features. For example, a psychometric score computed on a few individual answers to a questionnaire may carry the same predictive informational content as some or all of the individual answers. For binary classification tasks, as in this work, JADBio employs standard statistical models (ridge logistic regression), non-statistical linear models (linear SVM), and non-linear models (decision trees, random forests, polynomial and Gaussian SVM). The final model is produced by applying the best performing configuration on all data. Thus, no samples are lost to estimation of performance.

To identify the winning configuration and estimate its predictive performance in an unbiased way, JADBio uses appropriate out-of-sample protocols (ie, protocols that hide some of the data from a configuration) and then evaluate the corresponding model on the held-out samples. Specifically, for small sample sizes, JADBio uses a stratified, *N* repeated, *K*-fold cross validation on each configuration. *K*-fold cross validation is a common estimation procedure. It partitions the available samples to *K* folds. It then feeds to a given configuration all folds but one and produces a model, which is then applied on the held-out fold to estimate performance (eg, AUC). The performance estimation of the models produced by the given configuration is the average over all folds. JADBio actually repeats the *K-*fold procedure *N* times and averages out the performance estimate. Each repetition randomly repartitions the data to different folds. The purpose of repeating cross validation is to reduce the variance of the estimate due to the specific partitioning to folds. “Stratification” is a specific variant of cross validation where each fold is constrained to have a class distribution (ie, percentage of PTSD cases vs. controls) similar to the class distribution in the original dataset. Stratification has been shown to reduce the variance of the estimation. The choice of the estimation protocol and the values of *N* and *K* are selected by the AI system based on the data characteristics (eg, sample size, imbalance of the classes) and the user preferences.

Notice that JADBio does not report the cross-validated estimate of the winning configuration: When one tries numerous configurations, the cross-validated estimate of the winner is overly optimistic [36]. For example, if the winning configuration has a cross-validated AUC of 0.9 out of 1000 other configurations, then the true expected AUC is likely to be <0.9. To remove the optimism, as well as compute confidence intervals of predictive performance, JADBio applies a method called Bootstrap Bias Corrected Cross-Validation. In general, the estimates returned by JADBio are conservative. The theory, algorithms, and empirical evidence for the estimation protocols are described in detail by Tsamardinos et al [36].

### Modeling Procedure: Three Experiments to Test Performance Estimation, Model Transference, and Overall Prediction Accuracy

#### Experiment 1

First, we performed a computational experiment to ensure that JADBio’s predictive performance estimates are trustworthy. JADBio uses internally, theoretically, and empirically backed-up out-of-sample protocols to estimate performance of the final model, while adjusting for bias [36]. Nevertheless, it is still important to make sure the estimates of the system can be trusted in this particular type of data and problem. To this end, we initially combined the cohorts (cohorts 1 and 3 for 2.5-year PTSD prediction [cohort1&3_2.5] and cohorts 1 and 2 for 6.5-year PTSD prediction [cohort1&2_6.5]) and randomly split them into 5 subsets. Next, JADBio was used to train and evaluate models on four-fifths of the data and externally validate model performance on the remaining one-fifth of the data. We did this repeatedly to utilize all data subsets for training and validation, after which the performance achieved on the held-out fold was compared against the estimate returned by JADBio on the training folds.

#### Experiment 2

Second, we performed a computational experiment to establish transferability of the models (ie, test whether models trained on one cohort transfer [generalize] to another cohort). Specifically, we trained 2 models: one for PTSD status at 2.5 years after returning home and one for PTSD status at 6.5 years after returning home, both on data from cohort 1. We then tested their performance on cohort 3 and cohort 2, respectively.

#### Experiment 3

Third, we produced final models for each outcome and corresponding performance estimates from all available data. The reasoning behind the use of all available data was that, on average, the predictive performance of models increases with increased available sample size. Of course, this leaves no out-of-sample data to estimate the predictive performance; however, provided that experiment 1 was successful, the JADBio estimates can be trusted. In addition, provided that experiment 2 was successful, the model is likely to transfer to a new cohort and could potentially be employed in practice. More specifically, we again used the combined data sets (cohort1&3_2.5 and cohort1&2_6.5) to train models for each outcome. In addition, JADBio also performs feature selection during modeling. The selected features are the ones that enter the final models and provide psychological insight into the PTSD development.

#### Experiment 4 (Exploratory): Removal of Important Variables to Check Model Flexibility

While not part of the study aims, results from experiments 1-3 encouraged us to examine if the PTSD symptom level was the sole reason for the achieved prediction accuracy. Hence, we repeated experiment 3, but removed the total PTSD symptom score from the set of possible predictors. The purpose of this experiment was to test the robustness of JADBio in real-world screening situations, where some of the predictors can be missing. A desirable property of a screening method is to maintain predictive performance using available information.

## Results

### Experiment 1: Assessing the Quality of Out-of-Sample Performance Estimation

Results from the testing of JADBio performance estimates are displayed in Table 2. AUCs varied between 0.80 and 0.84 (mean 0.83) for the 2.5-year prediction and between 0.71 and 0.91 (mean 0.78) for the 6.5-year prediction. Importantly, the performances achieved on the validation sets are consistent with the JADBio performance estimates produced on the training sets; in fact, performance on the validation set was higher on average than the one estimated on the test sets. The results corroborate previous work [36], indicating that the estimation protocols within JADBio can be trusted on this data distribution. This implies that there is no need to reserve a separate hold-out set for estimating performance and lose samples to estimation.

**Table 2:** Areas under the receiver operating characteristic curves (AUCs) for the 5 training and test sets of the 2 cohorts.

Training-Testing set	2.5-year prediction		6.5-year prediction	
	Performance on the training set, AUC (95% CI)	Performance on test set, AUC	Performance on training set, AUC (95% CI)	Performance on test set, AUC
Training1-Testing1	0.77 (0.69-0.84)	0.82	0.77 (0.70-0.831)	0.73
Training2-Testing2	0.77 (0.68-0.84)	0.80	0.76 (0.69-0.83)	0.76
Training3-Testing3	0.73 (0.63-0.81)	0.84	0.74 (0.66-0.80)	0.91
Training4-Testing4	0.81 (0.73-0.88)	0.84	0.81 (0.74-0.87)	0.71
Training5-Testing5	0.78 (0.69-0.85)	0.82	0.74 (0.67-0.81)	0.79
Mean	0.77	0.83	0.76	0.78

### Experiment 2: Assessing Model Transferability to Different Cohorts

For the 2.5-year threshold, the AUC of the best-found model was estimated at 0.76 (95% CI 0.67-0.84), while the AUC on the external validation cohort 3 was 0.79. For the 6.5-year threshold, the AUC of the best-found model was estimated at 0.70 (95% CI 0.60-0.80), while the AUC on the external validation cohort 2 was 0.81. The results provide evidence that models trained on one cohort transfer to a future cohort. Obviously, care needs to be applied with this statement for cohorts that are obtained far apart in time, on totally different populations, and for different military conflicts.

### Experiment 3: Predictive Modeling for Potential Clinical Use

To produce the final predictive models for potential clinical use, we ran JADBio on cohort1&3 and cohort1&2 with PTSD status at 2.5 and 6.5 years, respectively, as the outcome. The user preferences were set to enforce feature selection and the analysis type to extensive, implying that a relatively large number of configurations would be explored. Overall, the analyses trained 450,200 and 417,800 models for the two outcomes, respectively, taking 32 and 40 minutes. All these models were trained using different configurations on different subsets of the data (cross validation) to estimate performance and produce a final optimal model. Detailed results from the prediction can be accessed in Multimedia Appendix 4.

**Figure 1:**
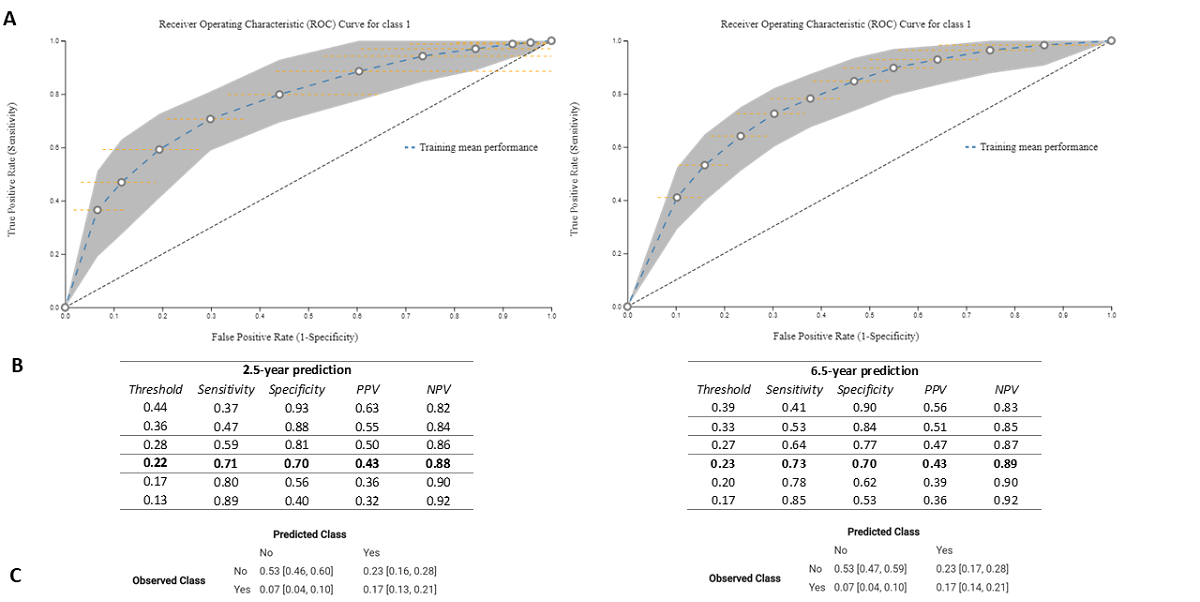
Results from the final prediction models (experiment 3): (A) receiver operating characteristic (ROC) curves for 2.5-year and 6.5-year predictions; (B) sensitivity, specificity, positive predictive value (PPV), and negative predictive value (NPV) for selected cutoffs on the ROC curve; (C) confusion matrices for selected cutoffs on the ROC curve (marked in bold in Panel B).

For the 2.5-year prediction, the optimal model was a random forest classifier trained with 100 trees and a minimal number of observations per leaf of 5 (AUC=0.77, 95% CI 0.71-0.83). For the 6.5-year prediction, the optimal model was also a random forest classifier trained with 1000 trees and a minimal number of observations per leaf of 5 (AUC=0.78, 95% CI 0.73-0.83). ROC curves as well as sensitivity, specificity, positive predictive value, and negative predictive value for selected cutoffs can be seen in Figure 1A and Figure 1B, along with a confusion matrix for a suggested balanced cutoff (Figure 1C). The results indicate that 2.5-year, as well as 6.5-year, prognosis of PTSD is possible with applicable levels of predictive accuracy.

### Experiment 3: Comparison Between Linear and Non-Linear Models

While random forests were the best overall performing models for both outcomes, JADBio also reported the best performing models out of those that are humanly interpretable. These were generalized linear models (ridge logistic regression) and decision trees. The best interpretable models for each outcome were both ridge logistic regression models. Their predictive performance was estimated to be indistinguishable from the random forests. The results show that, in these predictive tasks, non-linear models do not significantly improve predictive performance.

### Experiment 3: Feature Selection

**Figure 2:**
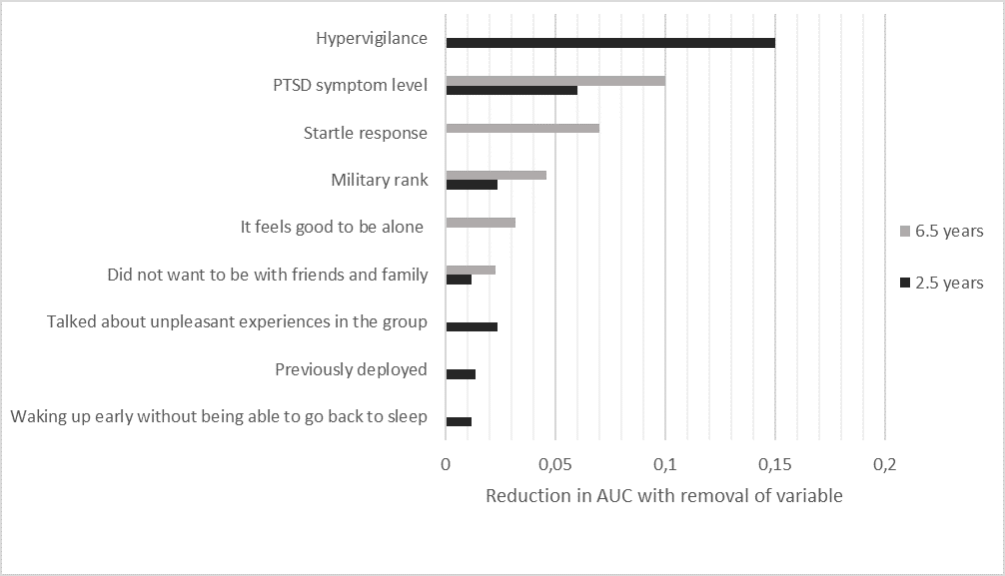
Cumulative variable importance for the two final prediction models. The x axis depicts the reduction in the area under the receiver operating characteristic curve (AUC) if the particular variable is removed from the model. The figure includes all selected variables resulting in an AUC reduction of at least 0.01 if removed. PTSD: posttraumatic stress disorder.

For the 2.5-year prediction, 4 similar, equally predictive feature sets were selected, each consisting of 14 features and, in total, including 16 different features across the 4 feature sets. For the 6.5-year prediction, a single, optimal feature subset was discovered consisting of 9 features (see Multimedia Appendix 5 for a total list of selected features). Variable importance is depicted in Figure 2, which includes all selected features of both models that lead to an AUC reduction of at least 0.01 if removed from the model. For both cohorts, total level of PTSD symptoms, military rank, and little desire to be with friends and family led to reductions in AUC if removed. Further, in both models, having a hyperarousal symptom had relatively large importance; for the 2.5-year prediction, hypervigilance showed the greatest importance, whereas for the 6.5-year prediction, a startle response led to a relatively large reduction in AUC if removed.

### Experiment 4 (Exploratory): Removal of Important Variables to Test Model Flexibility

For the 2.5-year as well as the 6.5-year prediction, the prediction accuracy of the models when removing the total level of PTSD symptoms remained the same (2.5-year prediction, AUC=0.77, 95% CI 0.71-0.82; 6.5-year prediction, AUC=0.78, 95% CI 0.72-0.84). The results indicate that, even when removing a central feature, predictive accuracy can be maintained.

## Discussion

For predictive models of PTSD symptomatology following military deployment to be useful in practical settings, several things are important: Applied models are not overly optimistic, predictive models built on current deployment cohorts can be transferred to future cohorts, and sufficiently high predictive accuracy can be reached for long-term PTSD outcomes (ie, several years following deployment). Testing and optimizing models manually are time-consuming; therefore, this study tested the applicability of AutoML as a means of enhancing model selection and parameter optimization. Hence, in the current study, we aimed to evaluate if this was achievable by combining data from 3 different cohorts deployed to Afghanistan with the Danish Defense between 2009 and 2013 to build predictive models using automated ML methods. Overall, we found that our applied AutoML software produced reliable estimates, the identified predictive models transferred well, and acceptable predictive accuracies were reached for prediction of screening-level PTSD 2.5 years after deployment (AUC=0.77, 95% CI 0.71-0.83) and 6.5 years after deployment (AUC=0.78, 95% CI 0.73-0.83). Further, we found that linear and nonlinear models performed equally well, and that, even with removal of one of the most central features, namely the total level of PTSD symptoms, screening-level PTSD at 2.5 years and 6.5 years could be predicted.

The use of an AutoML program such as JADBio warrants some discussion. A major advantage of using such a program is that it allows us to test multiple combinations of algorithms and their hyperparameter values within a reasonable time frame without need of extensive computer power. One drawback is that we have to rely on the settings of JADBio in performance evaluation. Here, one might worry that JADBio could be overestimating performance of identified models, for example by insufficient correction for the multitude of tested models [37]. To test if this was the case, in experiment 1, we performed a 5-fold cross validation of our two combined data sets where one-fifth of the data were repeatedly held out for external validation. Reassuringly, for all 10 validations, we found that the external validations of the test set revealed similar prediction accuracy as for the training set — all except three within the training set Cis, one slightly below, two slightly above. Hence, it seems reasonable to conclude that performance evaluation in JADBio is not overly optimistic, at least for the size and type of data examined here. This assured us that we can apply JADBio on all samples available for a given task without having to withhold a separate validation (hold-out) set and lose samples to estimation.

Having established that, model transferability was the next important prerequisite for the successful implementation of ML-based screening of deployment cohorts based on routinely collected data. Our results suggest that predictive models built on one cohort can indeed transfer to other cohorts. When predicting screening-level PTSD 2.5 years after deployment, our results showed that the model trained and tested on cohort 1 performed with similar accuracy in cohort 3 (cohort 3 AUC=0.79; cohort 1 AUC=0.76, 95% CI 0.67-0.84). When predicting screening-level PTSD 6.5 years after deployment, our results actually suggested that the model trained and tested on cohort 1 performed better on cohort 2 (cohort 2 AUC=0.81; cohort 1 AUC=0.70, 95% CI 0.60-0.80). Hence, for the deployment cohorts included in this study, it seems safe to say that models trained and tested on one cohort can transfer to another cohort. Importantly, while all cohorts included in this study deployed to Afghanistan, they did so at different times, with cohort 1 deploying in 2009 and cohort 3 in 2013. Conditions, tasks, threat levels, and deployment environments were similar between cohorts 1 and 2 [38] while substantially different between cohorts 1 and 3 [39], suggesting that even when deployment characteristics are not the same, predictive models can be transferred between cohorts deployed in similar missions. This is important given a wish to apply predictive models identified on existing data to future deployment cohorts.

This is one of the first few studies that tests how accurately PTSD can be predicted several years following deployment, more accurately, 2.5 and 6.5 years after returning home. From the literature, we know that symptoms of PTSD might develop with some delay following trauma, especially when the trauma occurs in an occupational context such as during military deployment [40,41]. Hence, predictive models trained to identify people who develop symptoms only during the first months following deployment might miss a great deal of those who go on to experience PTSD symptomatology. Our models predict screening-level PTSD at 2.5 years and 6.5 years with similar, acceptable accuracy (AUCs=0.77 and 0.78, respectively). Further, based on the model values of sensitivity, specificity, positive predictive value, and negative predictive value (Table 1), our findings show that we can achieve a reasonable balance for screening purposes. For example, with a sensitivity of 0.73 (for 6.5-year prediction), 89% of those who screen negative will indeed be noncases, while 43% of those who screen positive will indeed be cases. Optimally, our models would have higher overall accuracy; however, we utilized routinely collected data that were not collected with prediction in mind, and we were interested in testing how accurately prediction models trained on these data could predict screening-level PTSD. While the prediction accuracy is acceptable, it is far from perfect, and future endeavors should preferably include features that might increase accuracy.

From the total number of features, relatively small features sets were selected for both predictive models (14 features for 2.5-year prediction, 9 features for 6.5-year prediction). Some overlap in selected features was seen, with 3 features showing high cumulative importance in both cohorts: military rank, diminished interest in being with friends and family, and total level of PTSD symptoms 6 months after returning home. Neither of these are surprising; Lower rank has consistently been identified as a risk factor for PTSD following military trauma [42], low levels of perceived social support following trauma exposure is a known risk factor for PTSD [43], and early post-trauma levels of PTSD symptoms has also been found to predict PTSD later on [6].

Further, we found that a hyperarousal symptom is important in both cohorts: For the 2.5-year prediction, hypervigilance was the single most important feature, leading to a 0.15 reduction in AUC if removed, while for the 6.5-year prediction, startle response was among the most important features. While it is somewhat surprising that individual hyperarousal symptoms were selected as predictive features over and above the total level of PTSD severity, hypervigilance and startle response have been found to be central symptoms in PTSD in previous research [44,45]. This finding illustrates one of the benefits of using ML approaches for predictive modeling: In linear approaches, overlapping constructs would likely go undetected as significant, individual predictors of the outcome. The Bayesian Network approach for feature selection implemented in JADBio clearly allows for detection of such related predictors that are nonetheless individually related to the outcome [35].

An analyst facing predictive modeling tasks does not know whether interpretable, standard statistical linear models suffice or more complex, nonlinear, ML-based models are necessary to achieve optimal predictive performance. JADBio automatically tries both types of models and allows an analyst to compare them on equal grounds. An analyst can thus gauge whether the use of complex, nonlinear models is justified by the increase in predictive performance achieved. In our analyses, it seems that the linear models performed equally well for any practical purpose.

Also, we found that even when removing one of the most important predictive features, the total level of PTSD symptoms at 6 months, the prediction accuracy did not decrease. Hence, even when total symptom level is not available at 6 months, screening-level PTSD at 2.5 and 6.5 years can be predicted. This implies that even with a limited number of individual symptoms, personal characteristics, and demographic variables available, we will be able to identify those who have the highest risk of developing screening-level PTSD.

Our study has some limitations that should be noted. First, while we included 3 different cohorts, they are similar in that they all deployed to Afghanistan. Hence, to test if models transfer also when, for example, the deployment country differs, we will need to include cohorts who deployed to other conflict zones. However, as already argued, the cohorts differed in important ways. Second, the response rate to the questionnaire was approximately 65% across cohorts. We know from earlier analyses based on these cohorts that more individuals among the nonresponders may have more mental health problems [3]. While this introduces a risk of bias, this is also the reality if future screening efforts are to be based on this approach: For all Danish deployment cohorts since 1998, the response rate varies around 65%, so screening will be limited to those who responded to the questionnaire. Third, since the PRIM questionnaire is already being used for screening, individuals might have been offered treatment as a result of their responses to PRIM, which is another source of potential bias.

Despite these limitations, our study illustrates how screening of future deployment cohorts can be based on ML-based predictive models based solely on routinely collected questionnaire data. Importantly, we have demonstrated that models developed on routinely collected data on one cohort can be successfully transferred to predict screening-level PTSD in another cohort deployed to similar missions and that satisfactory predictive accuracy can be reached such that the model can be used as an actual decision support tool. In future efforts, we suggest that the models are further validated in cohorts deployed to other missions. For cohort 3 of this study, a follow-up collection of post-deployment data including measurement of PTSD symptoms is being conducted in the spring and summer of 2020. We predict that, based on a model trained on our routinely collected data at 6 months after homecoming for cohorts 1 and 2, we will be able to classify screening-level PTSD 6.5 years after returning home in cohort 3 with an AUC between 0.73 and 0.83. We intend to preregister and publish the results of this endeavor.

## Appendix

Multimedia Appendix 1 Full list of predictors.

Multimedia Appendix 2 Descriptive characteristics of all variables in cohorts 1 and 2.

Multimedia Appendix 3 Descriptive characteristics of all variables in cohorts 1 and 3.

Multimedia Appendix 4 Detailed results and visualizations from JADBio.

Multimedia Appendix 5 Selected features for 2.5-year and 6.5-year predictions.

## Abbreviations

AI: artificial intelligence.
AUC: area under the receiver operating characteristic curve.
AutoML: automated machine learning.
ISAF: International Security Assistance Force.
JADBio: Just Add Data Bio.
ML: machine learning.
PCL-C: civilian version of the PTSD checklist.
PRIM: Psychological Reactions to International Missions.
PTSD: posttraumatic stress disorder.
ROC: receiver operating characteristic.
SVM: support vector machine.
