# Supplementary material for: Applicability of an Automated Model and Parameter Selection in the Prediction of Screening-Level PTSD in Danish Soldiers Following Deployment: Development Study of Transferable Predictive Models Using Automated Machine Learning
Source: JMIR Med Inform. 2020 Jul 22;8(7):e17119. doi: 10.2196/17119 (PMC7407253; doi:10.2196/17119)
Supplement: Multimedia Appendix 1 [file medinform_v8i7e17119_app1.docx]

| SP1 | In which unit did you serve? |
| --- | --- |
| SP2 | Under which type of contract did you serve? |
| SP3 | What was your rank during your deployment? |
| SP4 | Did you previously deploy in international service? |
| SP5 | What is your age? |
| SP6 | What is you marital status? |
| SP7 | Do you have children? |
| SP8 | Did you return from the mission before planned? |
| SP9 | What has your employment status been since home-coming? |
| SP10.1 | Too little to do while in service |
| SP10.2 | Too little to do while off-duty |
| SP10.3 | Meaningless tasks |
| SP10.4 | Excessive workload |
| SP10.5 | Sudden changes in orders |
| SP10.6 | Dangerous situations |
| SP10.7 | A lack of private space |
| SP10.8 | Uncertainty in tasks |
| SP11 | Have you been injured/wounded during the deployment? |
| SP12 | Have you witnessed fellow soldiers being killed or wounded/injured? |
| SP13 | Have you been taken hostage or being withheld without consent? |
| SP14 | Have any of your fellow soldiers been taken hostage or withheld without consent? |
| SP15.1 | Being threatened with a weapon |
| SP15.2 | Being shot at |
| SP15.3 | Having fired your weapon (warning shots/in response to fire) |
| SP15.4 | Being under fire aimed at or close to your quarters |
| SP15.5 | Being in areas with roadside bombs or mines? |
| SP15.6 | Passing areas with combat activities |
| SP15.7 | Threats from participants of the conflicts |
| SP15.8 | Aggressive behavior from the locals |
| SP15.9 | Witnessing distress among the locals |
| SP15.10 | Seeing dead people |
| SP15.11 | Seeing wounded people |
| SP15.12 | Being witness to assaults on civilians |
| SP15.13 | That the participants of the conflict prevented you from performing your task |
| SP15.14 | Insufficient reinforcement or relief from your unit |
| SP16.1 | Being powerless |
| SP16.2 | Being humiliated or violated |
| SP16.3 | That your felt that situations were dangerous |
| SP16.4 | That you thought of the risk of being injured/killed yourself |
| SP16.5 | That you thought of the risk of a fellow soldier being injured or killed |
| SP16.6 | That it was difficult remaining neutral to the conflict |
| SP17.1 | That members of your group showed interest in one another |
| SP17.2 | That members of your group talked about unpleasant experiences |
| SP17.3 | That you received support and encouragement from superiors |
| SP18.1 | That you had worries about relatives at home |
| SP18.2 | That you had a good contact with relatives at home |
| SP18.3 | That you had trouble with relatives at home |
| SP19 | How often during your deployment did you experience situations as extremely threatening? |
| SP20 | How often during your deployment did you experience situations that were highly stressful emotionally? |
| SP21.1 | Being with friends and family did not appeal to me very much |
| SP21.2 | There was great interest among friends and family to hear about my experiences |
| SP21.3 | It was easy to get started with everyday life again |
| SP21.4 | There was great support from friends |
| SP22.1 | The home coming programme was well organized |
| SP22.2 | I received useful information from the home coming programme |
| SP22.3 | I received useful information on psychological responses after deployment |
| SP23.1 | Difficulties in taking care of your job or education |
| SP23.2 | Having gone through a divorce or the termination of a relationship |
| SP23.3 | Difficulties in balancing personal finances |
| SP23.4 | Having been physically injured |
| SP23.5 | Having experienced physical injury among close relatives |
| SP23.6 | Severe illness/death among close relatives |
| SP24.1 | That the relationship to my nearest relatives is good |
| SP24.2 | That it feels good to be with friends |
| SP24.3 | That once in a while it is good to be alone |
| SP24.4 | That I can share my concerns and problems with friends and family |
| SP25 | Have you been spending time with members of your deployment since the home coming gathering? |
| SP26 | Did you receive psychological assistance after home coming? |
| SP26a | From whom did you recieve assistance? |
| SP27.1 | Have achieved valuable military experience |
| SP27.2 | Have experienced good sense of fellowship |
| SP27.3 | Have achieved experiences of utility in civilian life |
| SP27.4 | Have learned about foreign contries/people |
| SP27.5 | Have widened your perspective |
| SP27.6 | Have acquired good friendships |
| SP27.7 | Have learned to overcome difficult situations |
| SP27.8 | Have achieved a stronger selfconfidence |
| SP27.9 | Believe that the deployment resulted in personal development |
| SP27.10 | Think of the deployment as a difficult period for you |
| SP27.11 | Believe that it was a demanding period for your partner/family/parents |
| SP27.12 | Can recommend to others to deploy |
| SP27.13 | Are proud of having served in international missions |
| SP28.1 | Trouble falling asleep |
| SP28.2 | nightmares |
| SP28.3 | headaches |
| SP28.4 | stomach problems |
| SP28.5 | easily gotten dizzy |
| SP28.6 | difficulties concentrating |
| SP28.7 | a bad conscience or feeling of guilt |
| SP28.8 | Were you easily saddened |
| SP28.9 | Did you have difficulties talking to others about your emotions |
| SP28.10 | Did you have difficulties in understanding others |
| SP29.1 | Disturbing thoughts or images, that suddenly appears |
| SP29.2 | a sense of unreality |
| SP29.3 | difficulties remembering parts of a stressful experience in the past |
| SP29.4 | difficulties remembering things |
| SP29.5 | difficulties controlling your temper |
| SP29.6 | trouble getting along with others |
| SP29.7 | a desire to hurt others |
| SP29.8 | a desire to avenge yourself |
| SP29.9 | worries about the future |
| SP29.10 | did you have thoughts about taking your own life |
| SP30.1 | did you wake up early in the morning – without being able to fall asleep again |
| SP30.2 | did you get very angry |
| SP30.3 | did you get so angry, that you wanted to “destroy everything” |
| SP30.4 | did you get so angry that you destroyed something |
| SP30.5 | involuntarily thoughts of a stressful experience in the past |
| SP31.1 | did you feel sad |
| SP31.2 | did you feel irritable |
| SP31.3 | did you feel lonely |
| SP31.4 | did you feel inferior or insecure |
| SP31.5 | did you feel empty inside |
| SP31.6 | did you feel abandoned |
| SP31.7 | been afraid |
| SP31.8 | felt isolated from others |
| SP32.1 | restless sleep |
| SP32.2 | did you worry a lot |
| SP32.3 | Felt that minor problems could bring you out of balance |
| SP32.4 | Been on guard towards others |
| SP32.5 | Tried to avoid others |
| SP32.6 | Did you feel that noone understood you |
| SP32.7 | Did you feel that something inside you is broken |
| SP32.8 | Did you feel that everything was meaningless |
| SP32.9 | Avoidance of things that could remind you of a stressful experience from the past |
| SP32.10 | Strong reactions to high, sudden noises |
| SP32.11 | Been afraid of walking outside paved roads/paths (on grass, gravel or similar) |
| SP33 | How would you evaluate your wellbeing today? |
| SP34 | When you think of the time prior to your deployment, how would you evaluate your wellbeing today? |
| PTSD_scale | PTSD - Total symptom score |
| PTSD_screen | Screening-level PTSD |
| PTSD_clin | Clinical-level PTSD |
| Trauma exposure | Exposed to trauma during deployment (Y/N) |
| Depression_scale | Depression – total symptom score |
| Depression_screen | Screening-level depression |
| Depression_clin | Clinical-level depression |
| Danger_exposure | Exposed to danger during deployment – scale |
| Danger_witnessing | Witnessing the consequences of war – scale |
| KOEN | Gender |
