# Supplementary material for: Applicability of an Automated Model and Parameter Selection in the Prediction of Screening-Level PTSD in Danish Soldiers Following Deployment: Development Study of Transferable Predictive Models Using Automated Machine Learning
Source: JMIR Med Inform. 2020 Jul 22;8(7):e17119. doi: 10.2196/17119 (PMC7407253; doi:10.2196/17119)
Supplement: Multimedia Appendix 4 [file medinform_v8i7e17119_app4.docx]

**Detailed results and visualizations from JADBio**

Detailed results from the 2.5-year prediction can be assessed in JADBio [here](https://app.jadbio.com/share/0417c8ad-12f4-4044-bc43-d772219d40f3) and from the 6.5-year prediction [here](https://app.jadbio.com/share/e730c992-d9c9-40e3-af2d-722b6f9aecdf).
