# Supplementary material for: Applicability of an Automated Model and Parameter Selection in the Prediction of Screening-Level PTSD in Danish Soldiers Following Deployment: Development Study of Transferable Predictive Models Using Automated Machine Learning
Source: JMIR Med Inform. 2020 Jul 22;8(7):e17119. doi: 10.2196/17119 (PMC7407253; doi:10.2196/17119)
Supplement: Multimedia Appendix 5 [file medinform_v8i7e17119_app5.docx]

**Table 2.** *Features selected for 6.5-year prediction, 2.5-year prediction, and for prediction of both outcomes.*

| **Prediction 6.5 years** | **Prediction 2.5 years** | **Prediction 2.5 years AND 6.5 years** |
| --- | --- | --- |
| Before/during deployment | Before/during deployment | Before/during deployment |
| - Witnessing assaults on civilians - Insufficient reinforcement or relief of the unit | - Previous deployments (Y/N) - Sudden changes in orders given - Felt that situations were dangerous | - Military rank |
|  | - In my group, we talked about unpleasant experiences - In my group, we showed interest for each other |  |
|  | - Felt that situations were extremely threatening |  |
|  | - Lack of privacy |  |
| After homecoming | After homecoming | After homecoming |
| - Strong reactions to sudden, high noises - It feels good to be alone for periods of time | - Divorce or termination of relationship - Having difficulties remembering | - Being on guard - Being with friends and family did not appeal to me very much |
|  | - Getting very angry in the months after deployment | - Level of PTSD-symptoms after deployment |
|  | - Trying to avoid other people | - Waking up early in the morning and not being able to go back to sleep |

***** *Selected among the top-three most predictive features in both outcomes.*
